# Prediction of overall survival in stage II and III colon cancer through machine learning of rapidly-acquired proteomics

**DOI:** 10.1038/s41421-024-00707-7

**Published:** 2024-08-13

**Authors:** Kailun Xu, Xiaoyang Yin, Hui Chen, Yuhui Huang, Xi Zheng, Biting Zhou, Xue Cai, Huanhuan Gao, Miaomiao Tian, Sijun Hu, Shu Zheng, Changzheng Yuan, Yongzhan Nie, Tiannan Guo, Yingkuan Shao

**Affiliations:** 1 Department of Breast Surgery and Oncology (Key Laboratory of Cancer Prevention and Intervention, China National Ministry of Education, Key Laboratory of Molecular Biology in Medical Sciences, Zhejiang, China), Cancer Institute, The Second Affiliated Hospital, Zhejiang University School of Medicine, Hangzhou, Zhejiang China; 2 Zhejiang Provincial Clinical Research Center for Cancer, Hangzhou, Zhejiang China; 3 Cancer Center of Zhejiang University, Hangzhou, Zhejiang China; 4 Department of Radiation Oncology, The Affiliated Hospital of Qingdao University, Qingdao, Shandong China; 5 School of Public Health, the Second Affiliated Hospital, Zhejiang University School of Medicine, Hangzhou, Zhejiang China; 6 School of Medicine, Westlake University, Hangzhou, Zhejiang China; 7 Westlake Center for Intelligent Proteomics, Westlake Laboratory of Life Sciences and Biomedicine, Hangzhou, Zhejiang China; 8 Research Center for Industries of the Future, School of Life Sciences, Westlake University, Hangzhou, Zhejiang China; 9 State Key Laboratory of Holistic Integrative Management of Gastrointestinal Cancers and National Clinical Research Center for Digestive Diseases, Xijing Hospital of Digestive Diseases, Fourth Military Medical University, Xi’an, Shaanxi China

**Keywords:** Colon cancer, Cancer models

**Fig. 1:**
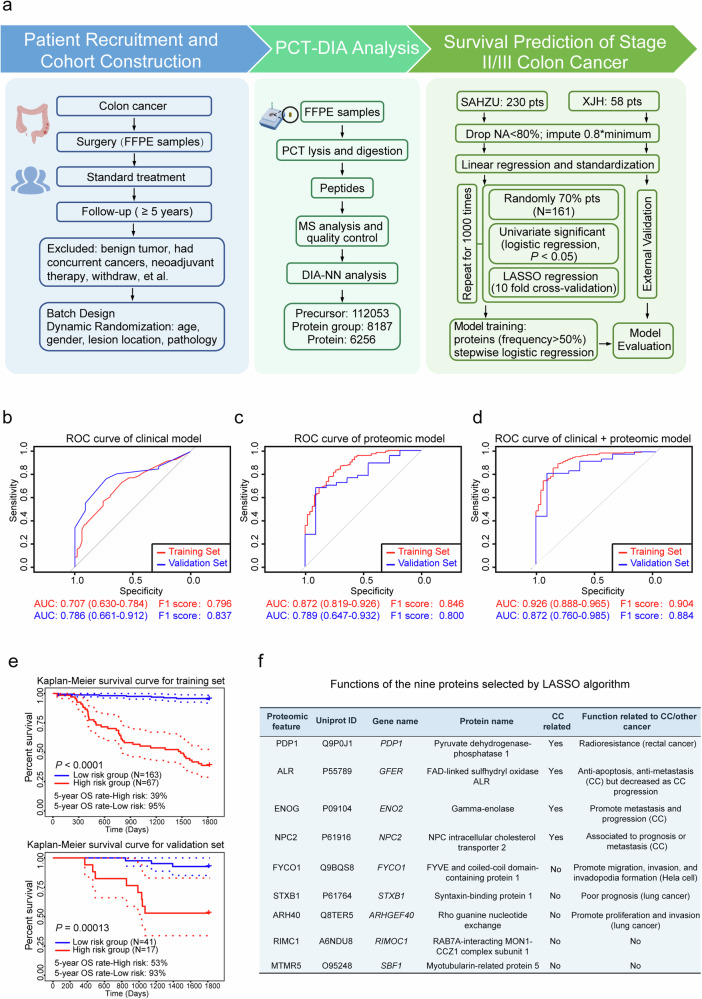
Schematic view of the study and performance of models. **a** Workflow for patient recruitment and cohort construction, PCT/MS analysis, and survival prediction of stage II–III CC. All the CC patients were followed up for over 5 years from SAHZU (*n* = 230) and XJH (*n* = 58) cohorts with strict criteria, and the FFPE samples were collected and designed into batches with dynamic randomization. Peptides extracted from the FFPE samples were quantified by MS analysis and determined with DIA-NN software. The SAHZU cohort was employed for model training with the LASSO regression; the model was then applied in the XJH cohort (validation cohort). **b** Receiver operating characteristic (ROC) curves of the clinical feature prediction model. **c** ROC curves of the proteomics prediction model. **d** ROC curves of the proteomics + clinical feature prediction model. AUC value with 95% confidence intervals (CI) and F1 score were listed for **b**–**d**. The F1 score is calculated as the harmonic mean of precision and recall. **e** Kaplan–Meier survival curve for the training set and the validation set. The 5-year OS rates were marked for the training set and the validation set, respectively. Log-rank test was used to calculate *P*-values. Dotted lines represent 95% CIs. **f** Known functions of the nine proteins selected by the LASSO algorithm.

Dear Editor,

Patients diagnosed with tumor-nodes-metastasis (TNM) stage II and III colon cancer (CC) account for over two-thirds of all CC cases. Clinicopathological patterns such as pT4 lesions (pathologically the tumor has grown into the surface of the visceral peritoneum or has attached to other organs or structures) and lymph node sampling < 12 nodes, as well as status of biomarkers *CDX2*, *SMAD4*, *BRAF*, and *KRAS*, are important factors that influence physicians’ choices regarding adjuvant treatment^1^. Patients with high-risk clinical features in stage II and those with stage III CC are typically advised to undergo adjuvant chemotherapy^2^. However, the universal applicability of adjuvant therapy for all stage III patients and the recurrence risk for other stage II patients is subject to ongoing debate^3^. Furthermore, existing risk factors does not accurately predict overall survival (OS)^4^, and other prognosis outcomes^5^, which calls for reliable prognostic markers or models to predict the prognosis of individual stage II–III CC patients. Such tools could enable more targeted treatment approaches for high-risk patients and prevent overtreatment of patients with an expected better prognosis. The aim of this study was to develop a comprehensible classification model to predict the long-term survival of stage II–III CC patients based on proteomics data and verify its generalizability in an external validation dataset. Here, we recruited patients with CC (stage II–III), all of whom underwent radical surgery and were followed up. Prior to the administration of any adjunctive treatments, we performed the proteomic analysis of formalin-fixed paraffin-embedded tissue (FFPE) surgical specimens using pressure cycling technology (PCT) and data-independent acquisition (DIA) mass spectrometry (MS)^6^. Leveraging machine learning algorithms, we established a novel and practical classification model for forecasting the prognosis in CC patients combining proteomic and clinical features, which was further verified in an independent validation cohort (Fig. 1a).

A total of 230 patients were recruited from the Second Affiliated Hospital of Zhejiang University (SAHZU) as the training cohort, and 58 patients were recruited from the Xijing Hospital (XJH) for external validation (Supplementary Table S1). All patients were followed up for over 5 years. We collected information on patients’ age, gender, lesion location, pathological type, stage, microsatellite instability (MSI) status (Supplementary Table S2) and built a clinical prognostic model using stepwise feature selection approach with the clinical features. Using PCT-DIA MS, a total of 8187 protein groups and 6256 proteins were identified and quantified in proteomic analysis with a high reproductivity (Supplementary Fig. S1a–f and Table S3). After 1000 replications of LASSO regression with resampled training set (Supplementary Fig. S2a), nine proteins were selected which were chosen in more than 50% times for proteomic model constructing, including PDP1, ALR, ENOG, NPC2, FYCO1, STXB1, ARH40, RIMC1, MTMR5 (Supplementary Fig. S2b, c and Table S4). We assessed the performances of this proteomic model, and the model combining the nine proteins with clinical features (lesion location, pathological type, stage, MSI status) to predict 5-year survival (yes or no) of stage II–III CC patients (Supplementary Table S5). In the training cohort, we improved the area under the receiver operating characteristic curve (AUC) value from 0.707 (clinical model) and 0.872 (proteomic model) to 0.926 (proteomic + clinical model). In the validation cohort, the AUC value was raised to 0.872 in the model incorporating clinical and proteomic data, from 0.786 in the clinical model and 0.789 in the proteomic model, respectively (Fig. 1b–d). Moreover, the sensitivity, specificity, positive predictive value (PPV), negative predictive value (NPV), overall accuracy and F1-score of the model combined with clinical and proteomic data were all elevated (Supplementary Table S6). Our model integrating clinical and proteomic data demonstrated a promising prognostic potential (Supplementary Fig. S2d), as evidenced by its ability to robustly stratify patients into low- and high-risk groups, with 5-year OS rates of 95% vs 39% in the training set (*P* < 0.0001), and 93% vs 53% in the validation set (*P* = 0.0013), respectively (Fig. 1e). The risk stratification was balanced (*P* > 0.05) regarding the use of adjuvant chemotherapy (Supplementary Table S7), which does not efficiently predict OS in the 5-year follow-up (Supplementary Fig. S3a).

Among the nine proteins, eight were downregulated in the patients surviving over 5 years and unfavorable for survival in CC, while only MTMR5 was upregulated and favorable for survival in CC (Supplementary Figs. S3b, 4a). The mRNA expression of *ENOG* from The Cancer Genome Atlas (TCGA) exhibited the similar result, and *NPC2* was further found to be unfavorable in MSI-high CC patients (Supplementary Fig. S4b). PDP1, ALR, ENOG and NPC2 have been implicated in CC progression (Fig. 1f). PDP1 activation may induce radioresistance in rectal cancer due to mitochondrial dysfunction^7^. ALR, as an anti-apoptotic and anti-metastatic factor, promotes cell survival and is involved in precancerous intestinal lesions^8^. ENOG promotes CC metastasis by epithelial-mesenchymal transition^9^ and was suggested to play a crucial role in the progression of *BRAFV600E*-mutated CC^10^. NPC2 functions as an intracellular cholesterol transporter and was found to contribute to prognosis and metastasis of CC^11^. FYCO1, STXB1, and ARH40 are involved in other tumors, but have not been reported in CC. Previous studies did not link MTMR5 and RIMC1 to tumors, which indicates the potential of our proteomics approach to unearth hidden essential proteins that are related to tumors. The function pathways related to *MTMR5* and *RIMC1* were discussed in the Supplementary Fig. S5a–c.

Several studies have developed novel approaches to improve the prognostication of TNM stage system, such as a six-microRNAs-based classifier for predicting CC recurrence in patients with stage II CC^12^ and a consensus immunoscore classification for stage I–III CC^13^. Combing MSI status, *BRAFV600E*, and *KRAS* mutation status with TNM staging improved the ability to precisely prognosticate in individual patients with stage II and III CC^14^. Additionally, deep learning allied to digital scanning of haematoxylin and eosin-stained sections have been reported to be employed in prognostic grouping for stage II–III CC^15^. However, the results of these methods were still not satisfactory enough to be widely adopted in clinical practice. In summary, we developed a novel clinical and nine proteins-based model to predict prognosis in stage II and III CC patients and validated it in an external cohort. Our model would assist in clinical decision-making by stratifying stage II and III CC patients. Patients at high-risk could be selected to receive more proactive treatment and follow-up, while those at low-risk could receive relatively low-level adjuvant therapy. Considering the limitations of this study, such as small sample size of the validation cohort, this model needs more validation and calibration in other independent cohorts. We are embarking on a clinical trial to prospectively test this model, with an aim to improve prognostication and aid in rational follow-up, schedule-making and risk-adaptive individualized therapies.

### Supplementary information

Supplementary information

Supplementary Table 1

Supplementary Table 3
