# Supplementary material for: Prediction of overall survival in stage II and III colon cancer through machine learning of rapidly-acquired proteomics
Source: Cell Discov. 2024 Aug 13;10:85. doi: 10.1038/s41421-024-00707-7 (PMC11319451; doi:10.1038/s41421-024-00707-7)
Supplement: Supplementary file 1 — Supplementary information [file 41421_2024_707_MOESM1_ESM.pdf]

Supplementary Information

Materials and Methods

Clinical samples and batch design

Written informed consent signed by all participants was based on the guidelines of the Declaration of Helsinki. Human tissue samples were collected with approval of the Institutional Ethics Committee of the Second Affiliated Hospital of Zhejiang University and Xijing Hospital of Digestive Diseases. A total of 334 colon cancer (CC) patients with stage I-IV from the Second Affiliated Hospital of Zhejiang University (SAHZU) in China from 2010-2012 were recruited previously. All patients were diagnosed and treated in accordance with national comprehensive cancer network (NCCN) guidelines, who underwent surgery with or without adjuvant therapy and followed up for up to 10 years. FFPE samples of 334 CC patients were punched by Manual Tissue Arrayer MTA-1 (Beecher, US) in equal size for pressure cycling technology (PCT)-DIA analysis (not published). The punches were checked to contain more than 70% tumor areas with the guidance of HE stained slides by two senior pathologists. We performed the batch design [1] to equally deposit samples obtained from individuals with different phenotypes to minimize batch effects for the large-scale sample preparation, including age, gender, lesion location and pathology with dynamic randomization algorithm. Each batch contains 15 CC samples, one mouse liver sample as quality control (QC) for PCT-based samples preparation method, and one CC pooled peptide sample as mass spectrometry (MS)-QC sample, as described previously [2].

The training cohort of 230 CC patients with stage II-III from SAHZU were included in the cohort mentioned above, and 58 patients were recruited by Xijing Hospital (XJH) for external validation (Supplementary table S1). Additional adjacent tissue cores from the same region were made as biological replicates to determine the extent of heterogeneity of CC samples, including 56 for SAHZU (24.3%) and 12 for XJH (20.6%).

Proteomic analysis

We performed PCT-DIA analysis of FFPE tissues as described previously [3]. After dewaxing, rehydration and hydrolysis of FFPE tissues (weight range: 0.8-1.0 mg), tissue was lysed in 6 M urea (Sigma-Aldrich) and 2 M thiourea (Sigma-Aldrich) assisted by PCT

at 30 °C for 90 cycles of 25 s at 45,000 p.s.i. and 10 s at ambient pressure (AP). After reduction and alkylation at atmosphere, proteins were first digested with lysC (Hualishi Scientific, China) assisted by PCT at a ratio of 1:40 (enzyme to substrate) at 30 °C using 45 cycles of 50 s at 20,000 p.s.i. and 10 s at AP. Tryptic digestion assisted by PCT was then performed with an enzyme-to-substrate ratio of 1:50 at 30 °C using 90 cycles of 50 s at 20,000 p.s.i. and 10 s at AP. Acidified peptides were desalted by Sep-Pak Vac 1cc (50 mg) C18 cartridges (Waters, MA, USA) following the manufacturer's protocol. For DIA acquisition, every 500 ng peptides of each sample were analyzed on a nano LC-MS/MS system (DIONEX UltiMate 3000 RSLC nano System, Thermo Fisher Scientific™, San Jose, USA) coupled to a Q Exactive HF Hybrid Quadrupole Orbitrap mass spectrometer (Thermo Fisher Scientific, San Jose, USA). Peptides were separated across a 60 min LC gradient (from 3% to 28% buffer B, 98%ACN, 2% H_2O containing 0.1% FA; buffer A was MS grade water containing 2 %ACN, 0.1% FA) at a flowrate of 300 nL/min. The time for sample loading was 4 min, while the time for washing and re-equilibration was 11 min. MS1 was acquired over a m/z range of 390-1010 with a resolution of 60,000 (at *m/z* 200), AGC target of 3e6, and maximum ion injection time (MIT) of 80 ms. Then 24 MS/MS scans were performed with a resolution of 30,000 (at *m/z* 200), AGC target of 1e6, MIT of auto and normalized collision energy of 28%. 66 and 12 randomly selected samples were acquired twice as technical replicates for SAHZU (28.6%) and XJH (20.6%) cohorts, respectively, to ensure high reproducibility of the study. All the DIA data of FFPE tissues were analyzed together by DIA-NN (version 1.7.4) [4] with the default parameters of mass accuracy and chromatogram scan window size, against a fasta file of Human proteins downloaded from https://www.uniprot.org/ on 9th Feb 2018, containing 222,038 reviewed entries. Our colorectal protein library, containing 118,608 peptides and 8,341 proteins, was built based on our DIA Pan-Human Library (DPHL) as previously reported [5]. The precursors were utilized to refine the pan-human library, resulting in the creation of colorectal protein subset library. The false discovery rate (FDR) was set to 1% at the peptide precursor level. And the resulting peptide matrix was converted to the protein matrix using ProteomeExpert server [6]. The final protein matrix used for downstream analyses was the abundance average of replicates from the same case, regardless of biological or technical replicates.

With PCT-DIA MS, a total of 6,256 proteins were identified and quantified in proteomic analysis. All the MS raw data in this report have been deposited to the ProteomeXchange Consortium via the iProX partner repository with the dataset identifier PXD048628.

Data preprocessing and model construction

Baseline characteristics, including age, sex, lesion location, pathological type, and MSI status, was employed for model building coupled with proteomic features. The participants survived over 5 years after recruitment was labelled as 'Y' group.

Firstly, we dropped 1,044 features with over 80% missing values proportion and the other 5,255 features were included in following modelling. Then proteins with missing values were imputed with 0.8 times the minimum value of the corresponding protein of other participants. We calculated the residuals of the linear regression models of proteins on age and sex and then standardize them (rank-based inverse normal transformation). For clinical model construction, the other four features (lesion location, pathological type, stage, microsatellite instability status) were employed as predictors using logistic regression. Then we selected proteins for proteomic model and combined model. We randomly selected 1000 seeds from 0~999999. With each seed, we randomly sampled 70% individuals from the training set to train a LASSO applied logistic regression with L1 regularization (LASSO, alpha =1, lambda chosen for minimal error) for feature selection from proteins that are significantly associated with the outcome (P < 0.05). We then selected the proteins that were selected >50% of the times for final proteomic model constructing. This approach of repeated modelling for feature selection procedure conferred a noteworthy element of stability to the result [7,8]. Proteins with frequency more than 50% were employed for model training with stepwise logistic regression. Nine proteins (PDP1, ALR, ENOG, NPC2, FYCO1, STXB1, ARH40, RIMC1, MTMR5) was selected, whose expression scattered among all proteins (Supplementary Fig. S2b), and all of them showed high quantification repeatability in the proteomic data (Supplementary Fig. S2c). And univariable and multivariable regression analysis confirmed the proteomics signature as an independent prognostic risk factor for II/III stage CC patients (Supplementary Table S8). Clinical features and proteomics combination model was evaluated using ROC curve

and AUC, and was compared with the models using clinical features or proteomics only. Resampling the training set and calculating the AUC for 1000 times was used to evaluate the stability of the model. The cutoff for the low-risk group was determined by the lowest probability with a positive likelihood ratio (LR+) > 5. Kaplan-Meier curve was employed to evaluate training set and validation set. Difference of selected proteins between patients survived for 5 years and those failed were calculated in the training set and validation set, respectively. The decision curve analysis (DCA) showed satisfactory predictive ability of proteomic + clinical model both in the training set and validation set (Supplementary Fig. S2d). In both cohorts, the blue line (model) was higher than the red line (all) and stays above the for a significant range of threshold probabilities despite nuances in thresholds. This suggests that using the model to guide treatment decisions leads to a great net benefit. We performed Kaplan-Meier survival analysis in the high and low-risk groups (Supplementary Fig. S3a), and found that neither in the high-risk nor low-risk group, there was no difference in OS time regardless of patients received chemotherapy or not. It might indicate that patients at high risk should receive more proactive treatment, while those at low risk could receive low-level adjuvant therapy or even be exempted from chemotherapy. Code was deposited in GitHub (https://github.com/YuanLabZJU/crca-proteome).

Protein function

Though FYCO1, STXB1, ARH40 have not yet been studied in the CRC, they were shown to be associated with migration, invasion, and poor prognosis in other tumors. FYCO1 was demonstrated to promote migration, invasion, and invadopodia formation in Hela cell [9]. The whole STXBP1 expression or membranal STXBP1 expression was correlated with poor prognosis and were independent prognostic factors of lung adenocarcinoma [10]. And ARH40 was reported to promote non-small cell lung cancer proliferation and invasion [11]. The remaining two proteins (MTMR5, RIMC1) has not been reported any association with tumor. MTMR5 acts as an adapter for the phosphatase MTMR2 to regulate MTMR2 catalytic activity and subcellular location and is related to Charcot-Marie-Tooth disease [12]. RIMC1, also named as C5orf51, plays an important role in the removal of damaged mitochondria via mitophagy by controlling the stability and localization of RAB family

GTPase RAB7A [13]. We profiled the gene expression profile of MTMR5 and RIMC1 across different tumor samples and paired normal tissues (Supplementary Fig. S5a). MTMR5 was down-regulated in the most tumors and RIMC1 was up-regulated. Relations between gene expression and abundance of tumor-infiltrating lymphocytes were analyzed (Supplementary Fig. S5b). MTMR5 expression was most significantly positively correlated with abundance of CD56dim natural killer cells and negatively correlated with abundance of effector memory CD4+ T cells, while RIMC1 showed the opposite trend (Supplementary Fig. S5c).

Statistical analysis

Statistical analysis was performed using R software (version 3.6.0). F1 score is used for measure of the model's balanced ability to both precision and recall from 0 to 1, where 1 is the best. Coefficient of variance (CV) was calculated as the ratio of the standard deviation to the mean for every protein among the mouse liver samples or pooled samples to evaluated the stability of MS. The sensitivity, specificity, positive predictive value (PPV), negative predictive value (NPV), and accuracy were calculated following the established methodology [14]. Chi-square test was used for nonparametric test of categorical data. Log-rank test was used to calculate *P*-values for Kaplan-Meier survival analysis. Student's *t*-test was used to calculate *P*-values in the expression of nine protein between patients survived over or less than 5 years in the training and validation sets, respectively. The overall survival analysis in Supplementary Fig. S4b and gene expression profile of MTMR5 and RIMC1 in Supplementary Fig. S5b analyzed on GEPIA (Gene Expression Profiling Interactive Analysis, http://gepia2.cancer-pku.cn/#index) [15], based on gene expression from the TCGA. Spearman correlations between expression of MTMR5 or RIMC1 and various lymphocytes across cancers analyzed by TISIDB (http://cis.hku.hk/TISIDB/) [16] based on mRNA data from the TCGA.

Reference:

1 Zhu, T. *et al.* BatchServer: A Web Server for Batch Effect Evaluation, Visualization, and Correction. *Journal of Proteome Research* **20**, 1079-1086, doi:10.1021/acs.jproteome.0c00488

(2020).

2 Sun, Y. *et al.* Artificial intelligence defines protein-based classification of thyroid nodules. *Cell Discov* **8**, 85, doi:10.1038/s41421-022-00442-x (2022).

3 Cai, X. *et al.* High-throughput proteomic sample preparation using pressure cycling technology. *Nature Protocols* **17**, 2307-2325, doi:10.1038/s41596-022-00727-1 (2022).

4 Demichev, V., Messner, C. B., Vernardis, S. I., Lilley, K. S. & Ralser, M. DIA-NN: neural networks and interference correction enable deep proteome coverage in high throughput. *Nat Methods* **17**, 41-44, doi:10.1038/s41592-019-0638-x (2020).

5 Ge, W. *et al.* Computational Optimization of Spectral Library Size Improves DIA-MS Proteome Coverage and Applications to 15 Tumors. *Journal of Proteome Research* **20**, 5392-5401, doi:10.1021/acs.jproteome.1c00640 (2021).

6 Zhu, T. *et al.* ProteomeExpert: a docker image based web-server for exploring, modeling, visualizing, and mining quantitative proteomic data sets. *Bioinformatics* **37**, 273-275, doi:10.1093/bioinformatics/btaa1088 (2021).

7 Krstajic, D., Buturovic, L. J., Leahy, D. E. & Thomas, S. Cross-validation pitfalls when selecting and assessing regression and classification models. *Journal of Cheminformatics* **6**, doi:10.1186/1758-2946-6-10 (2014).

8 Machado-Fragua, M. D. *et al.* Circulating serum metabolites as predictors of dementia: a machine learning approach in a 21-year follow-up of the Whitehall II cohort study. *BMC Medicine* **20**, doi:10.1186/s12916-022-02519-6 (2022).

9 Sun, X. *et al.* FYCO1 regulates migration, invasion, and invadopodia formation in HeLa cells through CDC42/N-WASP/Arp2/3 signaling pathway. *Biochem Cell Biol* **100**, 458-472, doi:10.1139/bcb-2021-0575 (2022).

10 Wang, X. *et al.* Membrane Location of Syntaxin-Binding Protein 1 Is Correlated with Poor Prognosis of Lung Adenocarcinoma. *Tohoku J Exp Med* **250**, 263-270, doi:10.1620/tjem.250.263 (2020).

11 Gu, J. *et al.* ARHGEF40 promotes non-small cell lung cancer proliferation and invasion via the AKT-Wnt axis by binding to RhoA. *Mol Carcinog* **61**, 1016-1030, doi:10.1002/mc.23457 (2022).

12 Kim, S.-A., Vacratsis, P. O., Firestein, R., Cleary, M. L. & Dixon, J. E. Regulation of myotubularin-related (MTMR)2 phosphatidylinositol phosphatase by MTMR5, a catalytically inactive phosphatase. *Proceedings of the National Academy of Sciences* **100**, 4492-4497, doi:10.1073/pnas.0431052100 (2003).

13 Yan, B.-R. *et al.* C5orf51 is a component of the MON1-CCZ1 complex and controls RAB7A localization and stability during mitophagy. *Autophagy* **18**, 829-840, doi:10.1080/15548627.2021.1960116 (2021).

14 Steward, D. L. *et al.* Performance of a Multigene Genomic Classifier in Thyroid Nodules With Indeterminate Cytology. *JAMA Oncology* **5**, doi:10.1001/jamaoncol.2018.4616 (2019).

15 Li, C., Tang, Z., Zhang, W., Ye, Z. & Liu, F. GEPIA2021: integrating multiple deconvolution-based analysis into GEPIA. *Nucleic Acids Research* **49**, W242-W246, doi:10.1093/nar/gkab418 (2021).

16 Ru, B. *et al.* TISIDB: an integrated repository portal for tumor-immune system interactions. *Bioinformatics* **35**, 4200-4202, doi:10.1093/bioinformatics/btz210 (2019).

Supplementary Fig. S1

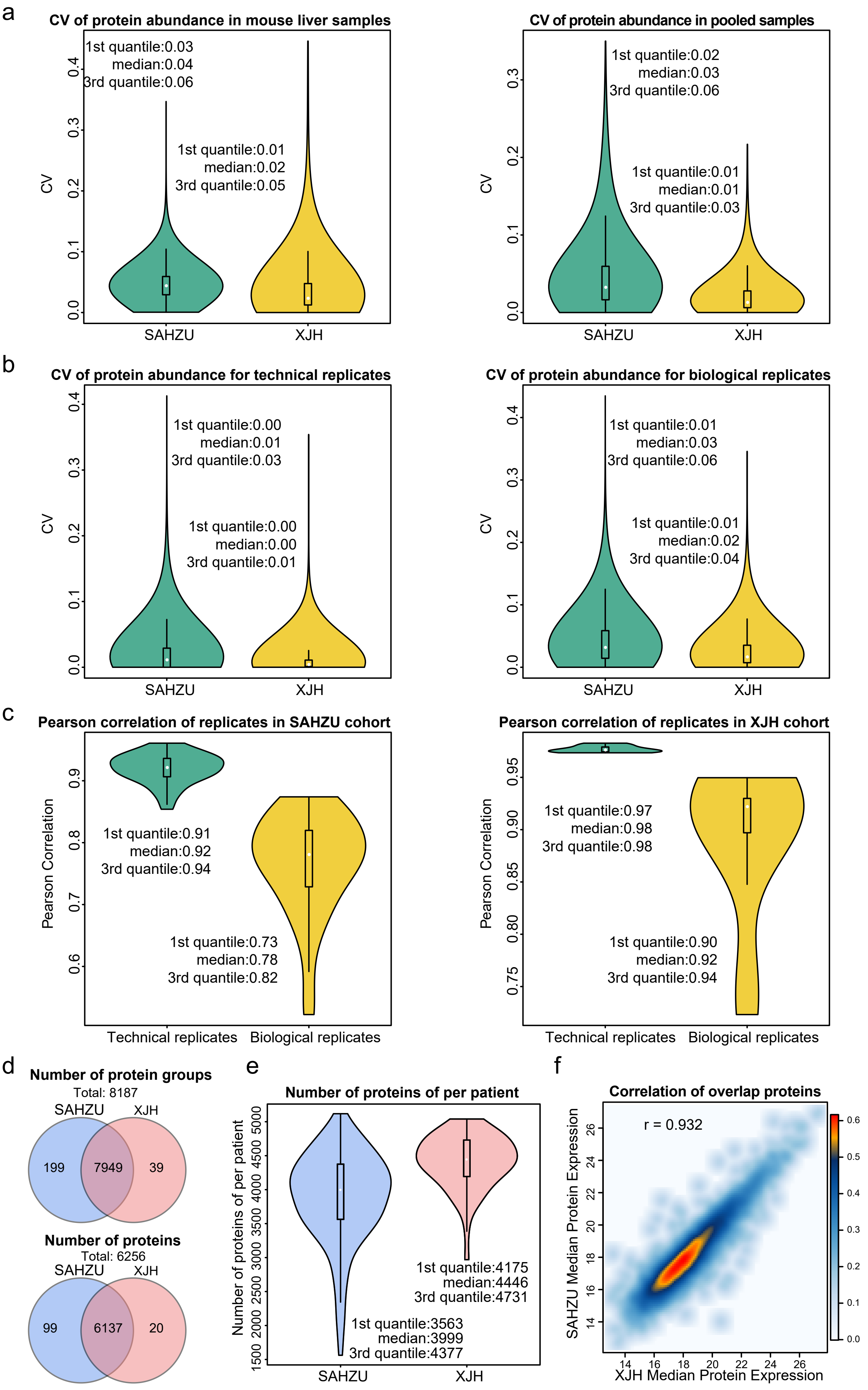

Supplementary Fig. S2

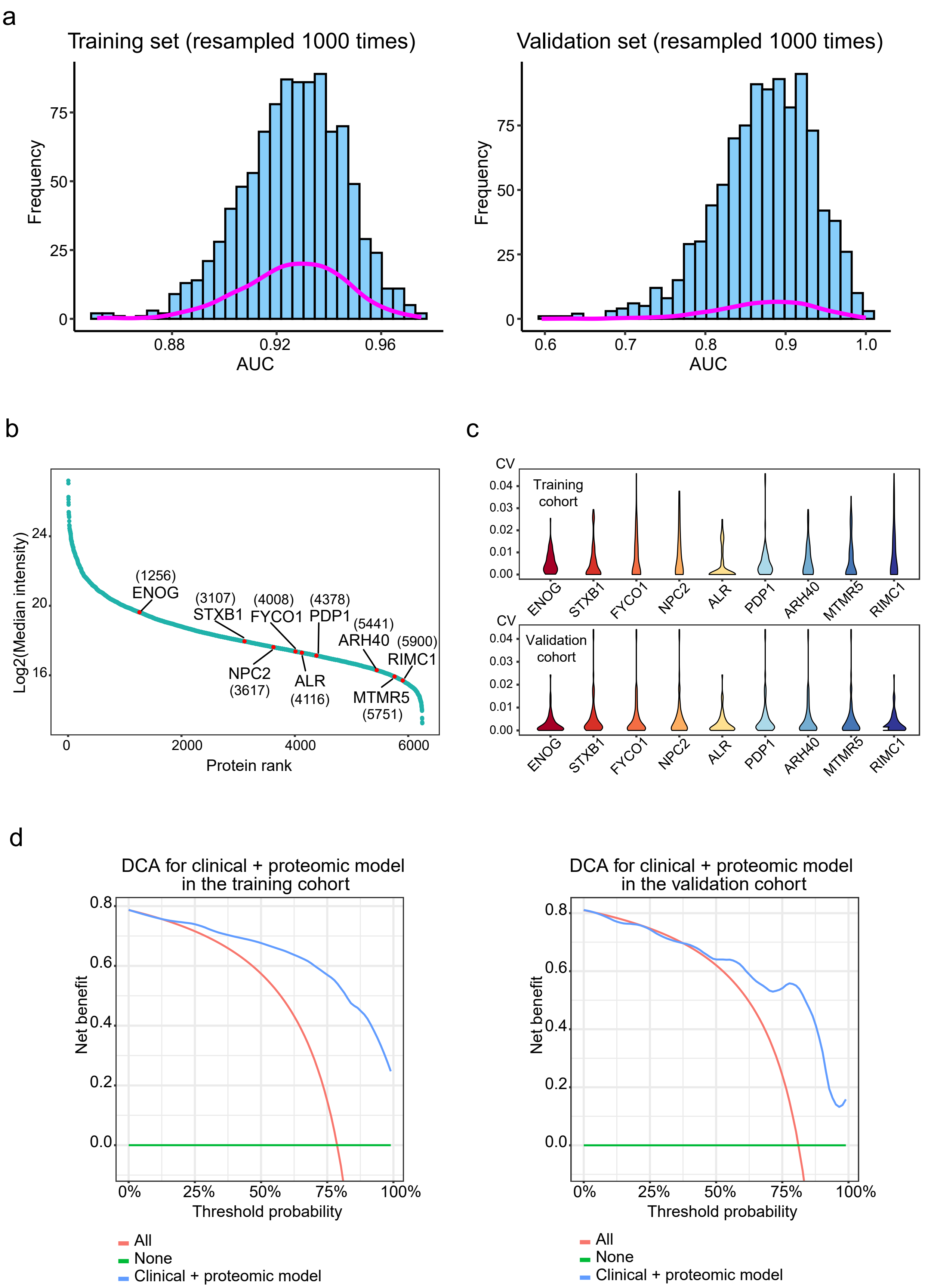

Supplementary Fig. S3

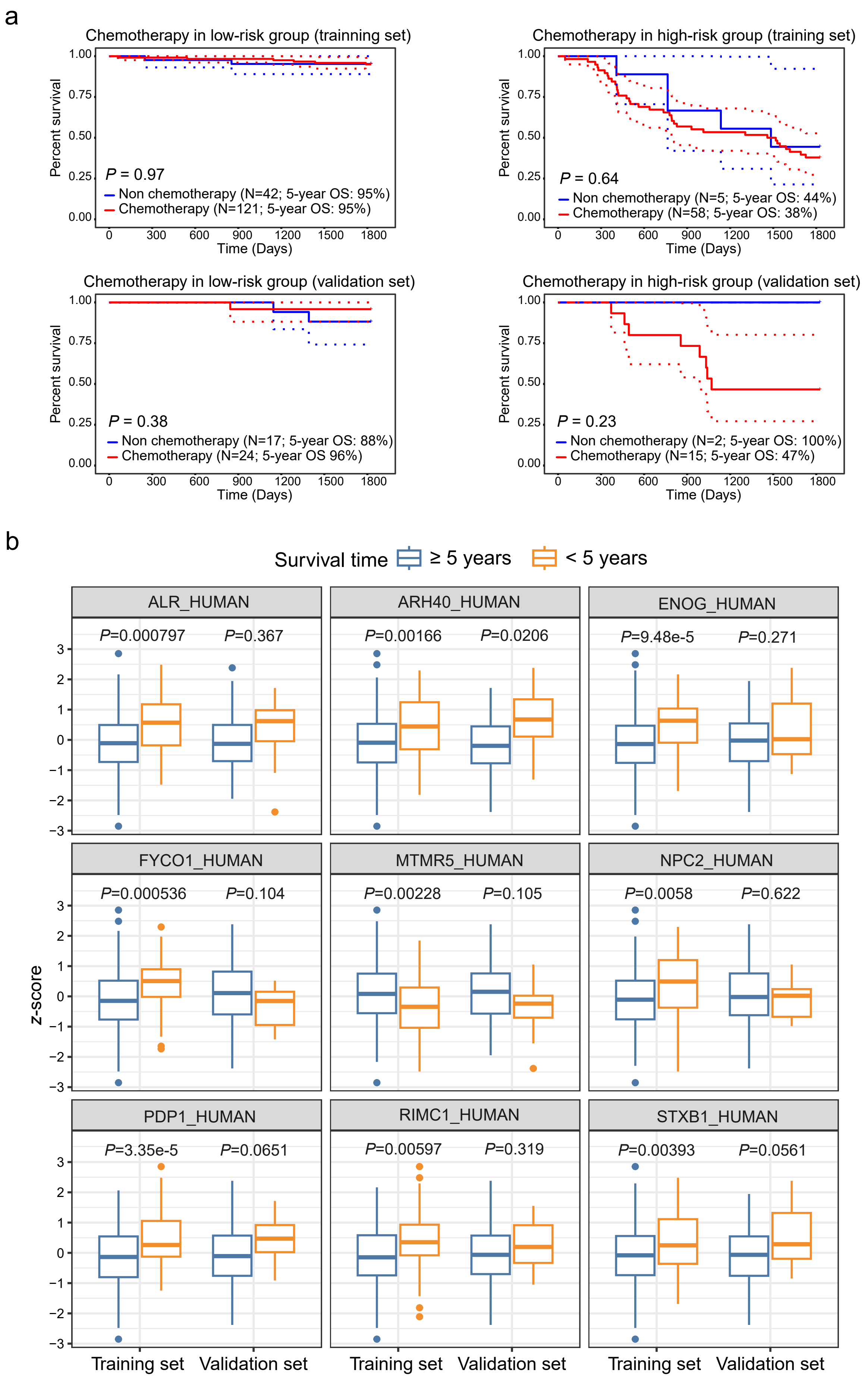

Supplementary Fig. S4

a

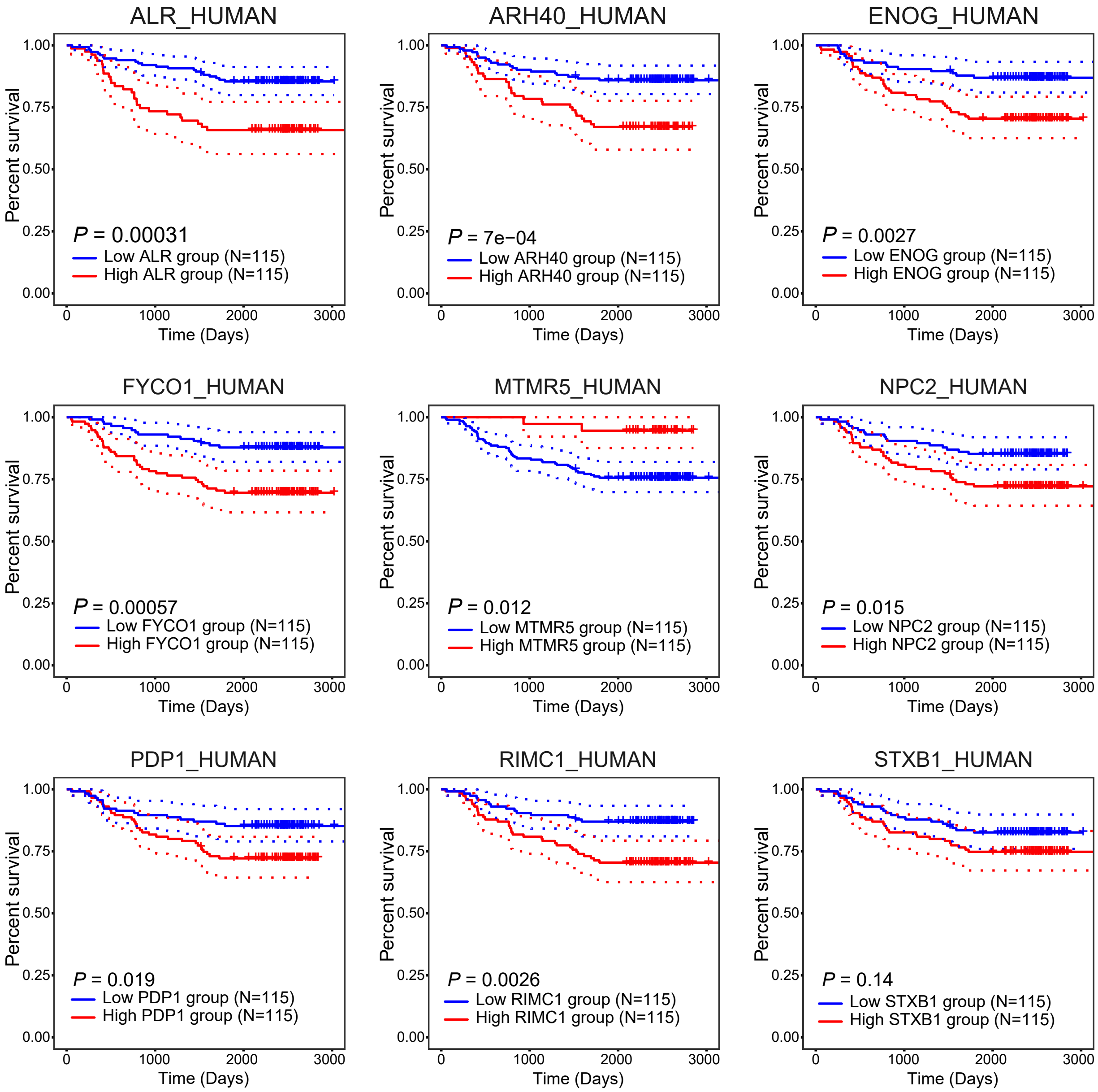

b

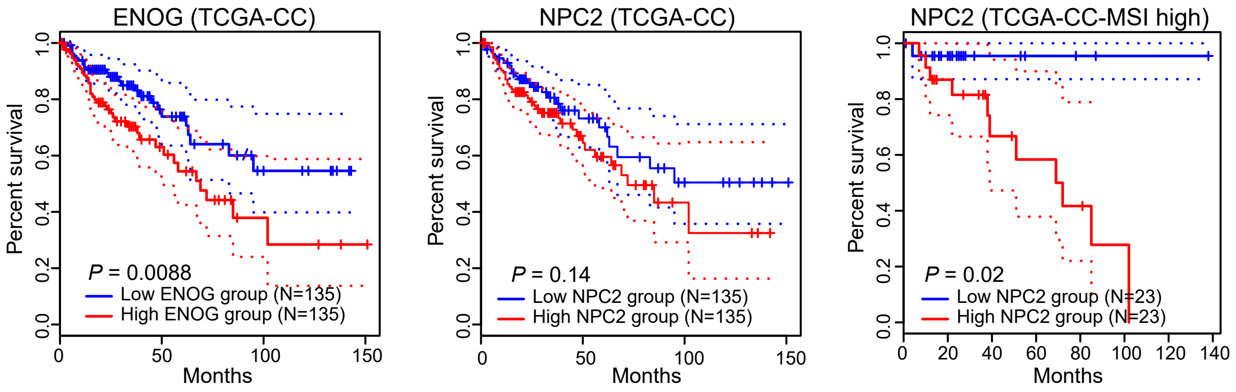

Supplementary Fig. S5

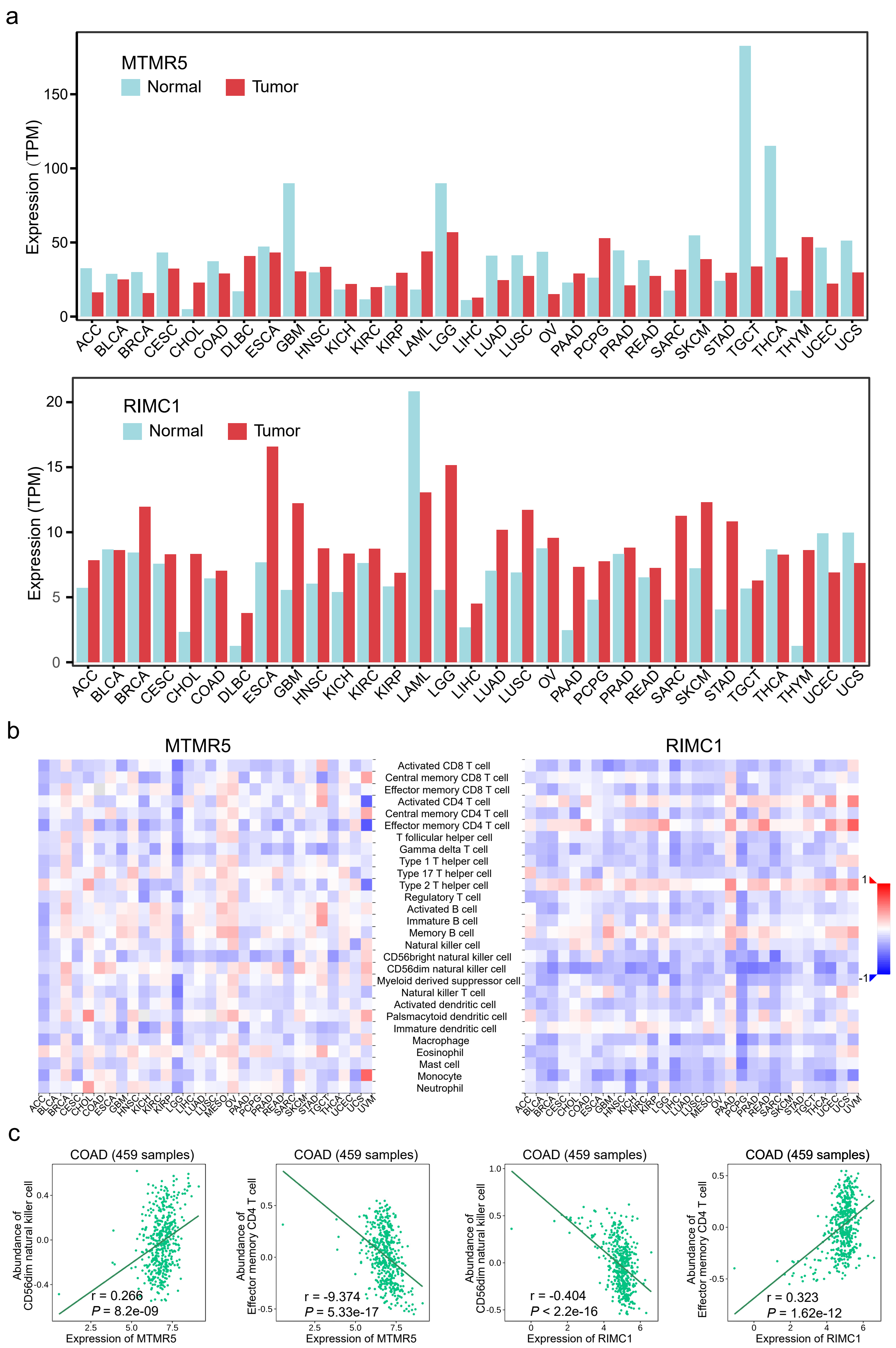

Supplementary Figure legends

Supplementary Fig. S1. Quality control of PCT-DIA MS data. a: Coefficient of variation (CV) of identified protein abundance for mouse liver and pooled samples. b: CV of identified protein abundance for technical replicates and biological replicates. For 1a-1b, CV was calculated as the ratio of the standard deviation to the mean for every protein among the samples. c: Pearson correlation of replicates in SAHZU and XJH cohorts. Values of −1 or +1 of Pearson correlation indicate a perfect linear relationship between the two variables. d: Venn diagrams exhibit overlap in identified protein groups and protein numbers between SAHZU and XJH cohorts. e: Number of proteins of per patient in the SAHZU and XJH cohorts. f: Overall Pearson correlation of overlap proteins in the SAHZU and XJH cohorts. The 1st quantile, median, and 3rd quantile value were shown in the 1a-1c and 1e.

Supplementary Fig. S2. a: Frequency histogram for 1000 AUC from 1000 subsets and 1000 resampled samples in the training and validation cohorts. The bars represent the frequency of a certain AUC value and the pink line was density curve representing the smooth distribution. The area under the curve is defined to be 1. b: Protein abundance distribution of the nine proteins. All proteins are ranked according to the log2(median intensity) from high to low. The rank of the nine proteins were marked. c: The CV distribution of technical replicates for the nine proteins in the training and validation cohort, respectively. d: The decision curve analysis (DCA) for proteomic + clinical model in the training and validation cohort, respectively. Net benefit = True positive rate – (False positive rate × Weighting factor). Weighting factor = Threshold probability / (1 – Threshold probability). The horizontal green line indicates that when no one survives over 5 years, no matter what the probability threshold is, the net benefit must be zero. The red line indicates that when everyone survives over 5 years, the net benefit changes as the probability threshold changes. And the blue line indicates the net benefit of the clinical + proteomic model.

Supplementary Fig. S3. a: Kaplan-Meier survival analysis showed overall survival of patients received chemotherapy or not in high and low-risk groups, respectively. Log-rank test was used to calculate *P*-values. Dotted lines represent 95% CI. b: Boxplots of expression of selected nine

proteins between patients who survived over 5 years and who survived less than 5 years in the training and validation sets, respectively. The expression of proteins was normalized by *z* score. Training set: N (survival time ≥ 5 years) = 180; N (survival time < 5 years) =50. Validation set: N (survival time ⩾ 5 years) = 47; N (survival time < 5 years) =11.

Supplementary Fig. S4. a: Kaplan-Meier plots showed overall survival of the 230 colon cancer patients from the SAHZU of the nine proteins. Samples with expression level higher than the median are considered as the high-expression group, otherwise the low-expression group. The dotted lines represent 95% CI. Log-rank test for hypothesis test. b: Kaplan-Meier plots showed overall survival of the colon cancer patients between low and high expression groups of ENOG and NPC2 analyzed by GEPIA (Gene Expression Profiling Interactive Analysis), based on mRNA data of CC from the TCGA. The median was used as the cutoff of the high- and low-expression group. The dotted lines represent 95% CI. Log-rank test for hypothesis test.

Supplementary Fig. S5. a: The bar plots showed gene expression profile of MTMR5 and RIMC1 across different tumor samples and paired normal tissues analyzed by GEPIA (Gene Expression Profiling Interactive Analysis), based on mRNA data from the TCGA. The height of bar represented the median expression of certain tumor type or normal tissue. b: The heatmaps of Spearman correlations between expression of MTMR5 or RIMC1 and various lymphocytes across cancers analyzed by TISIDB based on mRNA data from the TCGA. Abbreviations: ACC: Adrenocortical Carcinoma; BLCA: Bladder Urothelial Carcinoma; BRCA: Breast Invasive Carcinoma; CESC: Cervical and Endocervical Carcinoma; CHOL: Cholangiocarcinoma; COAD: Colon Adenocarcinoma; DLBC: Diffuse Large B-cell Lymphoma; ESCA: Esophageal Carcinoma; GBM: Glioblastoma Multiforme; HNSC: Head and Neck Squamous Cell Carcinoma; KIRC: Kidney Renal Clear Cell Carcinoma; KIRP: Kidney Renal Papillary Cell Carcinoma; LAML: Acute myeloid leukemia; LGG: Brain Lower Grade Glioma; LIHC: Liver Hepatocellular Carcinoma; LUAD: Lung Adenocarcinoma; LUSC: Lung Squamous Cell Carcinoma; MESO: Mesothelioma; OV: Ovarian Serous Cystadenocarcinoma; PAAD: Pancreatic Adenocarcinoma; PCPG: Pheochromocytoma and Paraganglioma; PRAD: Prostate Carcinoma; READ: Rectal Adenocarcinoma; SARC: Sarcoma; SKCM: Skin Cutaneous Melanoma; STAD: Stomach

Adenocarcinoma; TGCT: Testicular Germ Cell Carcinoma; THCA: Thyroid Carcinoma; UCEC: Uterine Corpus Endometrial Carcinoma; UCS: Uterine Carcinosarcoma; UVM: Uveal melanoma.

Supplementary Table 2. Clinical characteristics of the study cohorts

	Overall	Training Cohort	Validation Cohort	*P* -value
N	288	230	58	
Age (y, mean [SD])	60.8±12.9	62.3±12.3	54.7±13.6	**<0.001**
Male (%)	173 (60.1)	136 (59.1)	37 (63.8)	0.619
Lesion location (%)				**0.011**
Ascending	19 (6.6)	19 (8.3)	0 (0.0)	
Left	34 (11.8)	28 (12.2)	6 (10.3)	
Right	131 (45.5)	105 (45.7)	26 (44.8)	
Sigmoideum	83 (28.8)	58 (25.2)	25 (43.1)	
Transverse	21 (7.3)	20 (8.7)	1 (1.7)	
Pathological type (%)				**0.004**
Canalicular Adenocarcinoma	228 (79.2)	173 (75.2)	55 (94.8)	
Mucious adenocarcinoma	43 (14.9)	41 (17.8)	2 (3.4)	
Mixed	17 (5.9)	16 (7.0)	1 (1.7)	
Tumor stage (%)				**0.045**
2	4 (1.4)	4 (1.7)	0 (0.0)	
3	245 (85.1)	201 (87.4)	44 (75.9)	
4a	32 (11.1)	21 (9.1)	11 (19.0)	
4b	7 (2.4)	4 (1.7)	3 (5.2)	
Lymph stage (%)				0.788
0	163 (56.6)	128 (55.7)	35 (60.3)	
1a	41 (14.2)	34 (14.8)	6 (12.1)	
1b	33 (11.5)	28 (12.2)	5 (8.6)	
1c	12 (4.2)	8 (3.5)	4 (6.9)	
2a	26 (9.0)	21 (9.1)	5 (8.6)	
2b	13 (4.5)	11 (4.8)	2 (3.4)	
Stage (%)				0.62
II	163 (56.6)	128 (55.7)	35 (60.3)	
III	125 (43.4)	102 (44.3)	23 (39.7)	
MSI status (%)				0.138
MSI high	57 (19.8)	41 (17.8)	16 (27.6)	
MSS	231 (80.2)	189 (82.2)	42 (72.4)	
Survival 5 years (%)	228 (79.2)	181 (78.7)	47 (81.0)	0.833

Notes： 1. Tumor stage and Lymph stage are items in the Tumor, node and metastasis (TNM) staging.

2. "T" plus number (0 to 4) is used to describe how deeply the primary tumor has grown into the bowel lining. T2: The tumor has grown into the muscle layer of the bowel wall. T3: The tumor has grown into the outer lining of the bowel wall but has not grown through it. T4a: The tumor has grown into the surface of the visceral peritoneum; T4b means the tumor has grown through the bowel wall into nearby organs.

3. Stage combines the information of Tumor, node and metastasis. Stage II: The cancer has grown through the wall of the colon, or the tumor has grown into the surface of the visceral peritoneum, or has attached to other organs or structures, but has not spread to nearby tissue or to the nearby lymph nodes (T3-4, N0, M0). Stage III: The cancer has spread beyond the lining of the colon to nearby lymph nodes but has not spread to distant part of the body (any T, N1-2, M0).

Supplementary Table 4. Coefficients of the proteomics prediction model

	Training cohort			Validation cohort			Coefficient (95%CI)	*P* -value
	Log2(Fold change)	*P* -value	Change	Log2(Fold change)	*P* -value	Change		
Intercept							2.16 (1.65, 2.76)	<0.001
PDP1_HUMAN	-0.6051	0.0001202	Down	-0.6626	0.228322	NotSig	-0.76 (-1.24, -0.33)	0.001
ALR_HUMAN	-0.9896	0.0015864	Down	-0.7252	0.239599	NotSig	-0.47 (-0.90, -0.06)	0.028
ENOG_HUMAN	-0.4488	0.0018305	Down	-0.5799	0.215364	NotSig	-0.84 (-1.33, -0.39)	<0.001
FYCO1_HUMAN	-0.6171	0.0024926	Down	0.5707	0.064105	NotSig	-0.40 (-0.85, 0.04)	0.076
STXB1_HUMAN	-0.8008	0.0179118	Down	-1.4165	0.200534	NotSig	-0.32 (-0.74, 0.08)	0.127
ARH40_HUMAN	-0.9472	0.0012516	Down	-0.9652	0.005571	Down	-0.53 (-0.97, -0.11)	0.015
RIMC1_HUMAN	-0.7752	0.008747	Down	-0.2191	0.281093	NotSig	-0.32 (-0.78, 0.12)	0.156
NPC2_HUMAN	-0.7488	0.0020078	Down	0.3192	0.325019	NotSig	-0.68 (-1.15, -0.25)	0.003
MTMR5_HUMAN	1.5198	0.004016	Up	0.3958	0.093233	NotSig	1.00 (0.53, 1.51)	<0.001

Supplementary Table 5. Coefficients of the model combined with proteomics data and clinical features

	**Coefficient (95%CI)**	**OR (95%CI)**	***P*-value**
Intercept	6.02 (3.48, 9.31)	412.49 (32.45, 11040.48)	<0.001
Lesion location			
Ascending	0 (ref.)	1 (ref.)	
Left	-0.09 (-3.17, 2.63)	0.92 (0.04, 13.89)	0.951
Right	-1.89 (-4.80, 0.23)	0.15 (0.01, 1.26)	0.132
Sigmoideum	-2.39 (-5.33, -0.25)	0.09 (0.00, 0.78)	0.06
Transverse	-1.51 (-4.65, 1.08)	0.22 (0.01, 2.94)	0.284
Pathological type			
Canalicular Adenocarcinoma	0 (ref.)	1 (ref.)	
Mucious adenocarcinoma	-1.17 (-2.44, 0.11)	0.31 (0.09, 1.11)	0.069
Mixed	0.93 (-0.98, 3.19)	2.54 (0.38, 24.31)	0.364
Stage			
II	0 (ref.)	1 (ref.)	
III	-1.67 (-2.73, -0.69)	0.19 (0.06, 0.50)	0.001
MSI status			
MSI-high	0 (ref.)	1 (ref.)	
MSS	-1.10 (-2.62, 0.22)	0.33 (0.07, 1.24)	0.122
PDP1_HUMAN	-0.75 (-1.31, -0.25)	0.47 (0.27, 0.78)	0.006
ALR_HUMAN	-0.77 (-1.31, -0.28)	0.46 (0.27, 0.75)	0.003
ENOG_HUMAN	-1.09 (-1.70, -0.56)	0.34 (0.18, 0.57)	<0.001
FYCO1_HUMAN	-0.66 (-1.22, -0.13)	0.52 (0.29, 0.88)	0.017
STXB1_HUMAN	-0.18 (-0.67, 0.31)	0.84 (0.51, 1.36)	0.479
ARH40_HUMAN	-0.74 (-1.28, -0.25)	0.48 (0.28, 0.78)	0.005
RIMC1_HUMAN	-0.39 (-0.90, 0.11)	0.68 (0.41, 1.11)	0.127
NPC2_HUMAN	-0.59 (-1.11, -0.09)	0.55 (0.33, 0.91)	0.022
MTMR5_HUMAN	1.24 (0.67, 1.88)	3.44 (1.96, 6.57)	<0.001

Supplementary Table 6. Performance of the three models

	Clinical model		Proteomic model		Clinical + proteomic model	
	Training cohort	Validation cohort	Training cohort	Validation cohort	Training cohort	Validation cohort
AUC	0.655 (0.575-0.735)	0.748 (0.614-0.881)	0.872 (0.819-0.926)	0.789 (0.647-0.932)	0.926 (0.888-0.965)	0.872 (0.760-0.985)
Sensitivity	0.633	0.727	0.796	0.909	0.857	0.909
Specificity	0.674	0.681	0.773	0.681	0.856	0.809
PPV	0.878	0.907	0.928	0.924	0.956	0.953
NPV	0.332	0.369	0.506	0.637	0.618	0.675
Accuracy	0.642	0.718	0.791	0.866	0.857	0.89

Supplementary Table 7. Risk Stratification of Adjuvant chemotherapy with clinical + proteomic model

Adjuvant chemotherapy (%)		No	Yes	*P* value
Training cohort	All (%)	51 (22.2)	179 (77.8)	0.061
	Low risk (%)	42 (25.8)	121 (74.2)	
	High risk (%)	9 (13.4)	58 (86.6)	
Validation cohort	All (%)	19 (32.8)	39 (67.2)	0.059
	Low risk (%)	17 (41.5)	24 (58.5)	
	High risk (%)	2 (11.8)	15 (88.2)	

Supplementary Table 8. Hazard ratios (HRs) and 95% confidence intervals (CIs) of mortality of high- versus low- risk groups defined according to proteomics signature

		Low-risk group (HR)	High-risk group (HR)
Training cohort	Univariable Model	1 (ref.)	18.64 (8.71, 39.90)
	Multivariable Adjusted Model 1	1 (ref.)	16.30 (7.29, 36.45)
	Multivariable Adjusted Model 2	1 (ref.)	16.20 (7.15, 36.72)
Validation cohort	Univariable Model	1 (ref.)	8.71 (2.30, 32.99)
	Multivariable Adjusted Model 1	1 (ref.)	6.69 (1.27, 35.25)
	Multivariable Adjusted Model 2	1 (ref.)	6.21 (1.24, 31.00)

Multivariable Adjusted Model 1: adjusted for lesion location, pathological type, stage, and MSI status
Multivariable Adjusted Model 2: adjusted for age, sex, lesion location, pathological type, stage, and MSI status
